# Changes in sleep patterns in adolescents are more associated with pubertal indicators than age: A perfect storm with a dash of hormones

**DOI:** 10.1111/bjdp.70028

**Published:** 2025-11-18

**Authors:** Yessica Alejandra Martínez‐Sánchez, Debora Cristina Hipolide, Rafaella Sales de Freitas, Sabine Pompeia

**Affiliations:** ^1^ Department of Psychobiology Universidade Federal de São Paulo São Paulo Brazil

**Keywords:** adolescents, circadian rhythms, evening trend, growth and development, puberty

## Abstract

As they become older, adolescents tend to prefer sleeping and rising later. Yet, it is still unclear if these sleep changes occur due to advancing age or because adolescents are more pubertally mature. This was investigated cross‐sectionally in a sample of 121 Brazilian 9‐to‐17‐year‐olds. Participants reported their sleep habits (bed/rise times, time in bed [TIB] on weekdays and weekends, social jetlag) and circadian preferences (Morningness–Eveningness Scale for Children), which were analysed according to changes in age and pubertal status [self‐rated Pubertal Development Scale (PDS); clinician‐rated Tanner stages]. Older age related only to later weekday bedtimes. Later week/weekend bedtimes and reduced TIB were more associated with Tanner stages, especially pubic hair (adrenal) maturation, while evening preference was linked to higher PDS and genital/breast (gonadal) Tanner stages. Adolescents' sleep habits mapped differently onto age/pubertal status: higher adrenal development related to reduced sleep time, while higher gonadal development associated with eveningness preferences.

## INTRODUCTION

Adolescents' well‐being is shaped by many factors including adapting to changes in their sleep patterns, which are partly due to psychosocial issues like social media use at bedtimes, lower parental monitoring of sleep onset, higher academic burden and earlier school start times (e.g., Chen et al., 2024; Crowley et al., 2018; Gariepy et al., 2020). Alterations in sleep schedules during adolescence additionally arise from physiological maturation, including circadian advancement of sleep phase, that is, a preference for falling asleep and waking later (Carskadon et al., 1993; Díaz‐Morales & Escribano, 2015; Roenneberg et al., 2004; Thorleifsdottir et al., 2002), although there are also individual differences in circadian preferences in this (de Santana et al., 2025) and other phases of life, which can be accounted for by genetic (Hur, 2007) and multiple environmental factors (Hagenauer & Lee, 2013). Compared to children, from the early stages of adolescence, sleep pressure accumulation processes over the wake period decelerate (slower buildup of homeostatic sleep pressure), making it easier for adolescents to delay their bedtimes (Crowley et al., 2018; Hagenauer & Lee, 2013). In contrast, the rate at which sleep pressure is dissipated during sleep only becomes as efficient as in adulthood later on (Crowley et al., 2018; Hagenauer & Lee, 2013) so that there is only a slight progressive decrease in total sleep time throughout adolescence (Gariepy et al., 2020; Gradisar et al., 2011; Olds et al., 2010).

Together, the abovementioned psychosocial, circadian and homeostatic changes observed during the second decade of life create a perfect storm for sleep problems (Crowley et al., 2018), making some adolescents prone to suffer from sleep deprivation (Crowley et al., 2018; Gariepy et al., 2020). This also leads adolescents, on average, to tend to compensate for sleep loss on free days (Gariepy et al., 2020), creating a difference in sleep schedules during weekdays and weekends that is termed social jetlag (Wittmann et al., 2006).

All the abovementioned sleep pattern changes impact academic achievement and are associated with lower immunity, increased odds of being overweight and less physically active, having difficulty dealing with emotions, lower well‐being and worse quality of life (e.g., Gariepy et al., 2020; Henderson et al., 2019). Consequently, it is crucial to better understand sleep changes in adolescence to identify those who are more vulnerable to these effects. It is also important to develop adequate interventions to circumvent these problems.

Most studies on sleep during adolescents describe changes in sleep patterns according to chronological age (e.g., Gariepy et al., 2020; Gradisar et al., 2011; Olds et al., 2010) as laid out so far. However, most publications ignore maturational changes that derive from pubertal development, a set of physiological processes triggered by increases in the production of gonadal (e.g., testosterone, oestrogen, progesterone) and certain adrenal hormones (e.g., dehydroepiandrosterone: DHEA), which occur independently but at a relatively similar pace (Dorn et al., 2006; Joos et al., 2018).

This myriad of neuroendocrine modifications alters outward physical appearance which begins (pubertal timing), in girls, from the ages of 8 to 13 and lasts on average four and a half years, but can progress (pubertal tempo) till completion in less than two, to up to 6 years (Joos et al., 2018). In boys the same occurs, but roughly one to 2 years later than in girls (Joos et al., 2018). Hence, age is not a good indicator of pubertal status (Dorn et al., 2006, 2019). These pubertal changes also relate to alterations in sleep physiology (Campbell et al., 2012; Crowley et al., 2014; Hagenauer & Lee, 2012, 2013; Lucien et al., 2021). Accordingly, compared to age‐matched peers, adolescents whose pubertal status is more advanced or delayed may be at different risks of developing sleep‐related problems and their negative consequences.

The gold standard in pubertal staging is assessment by physical examination carried out by experienced clinicians using the Tanner scale (Dorn et al., 2006, 2019) which classifies individuals into one of five stages of puberty in regard to pubic hair growth, indicative of adrenal maturation and breast (girls) and genital (boys) characteristics, which index gonadally induced changes (see Dorn et al., 2006, 2019). However, the most common means of determining pubertal status, especially in the sleep literature, is self‐assessment using the Pubertal Development Scale (PDS: e.g., Carskadon et al., 1993; Petersen et al., 1988), which enquires about a combination of adrenally and gonadally induced secondary sexual characteristics. PDS scores are considered less reliable than Tanner scores (Campisi et al., 2020), although they do exhibit predictive and discriminant validity (e.g., Hibberd et al., 2015; Shirtcliff et al., 2009).

Higher PDS scores have been found to relate to later bedtimes and evening preferences (Borrani et al., 2020; Carskadon et al., 1993; Diao et al., 2020; Díaz Morales et al., 2023; Randler et al., 2009), which may be due to a delayed circadian phase and/or to lowering of the buildup of homeostatic sleep pressure, as both contribute to later sleep onset (Crowley et al., 2018). PDS scores indicative of higher pubertal status have additionally been associated with shorter sleep duration on school nights (Diao et al., 2020; Mozafarian et al., 2023; Randler et al., 2009; but not in Borrani et al., 2020), which is indicative of more efficient dissipation of sleep pressure across a night's sleep (Crowley et al., 2018).

Similar changes have been observed regarding Tanner pubic hair ratings, which indicate adrenal maturity, scores being positively related to later bedtimes on weekdays or weekends (e.g., Crowley et al., 2014; Foley et al., 2018), markers of circadian phase delay (phase of melatonin secretion: Carskadon et al., 1997, 2004; later dim light melatonin onset: Crowley et al., 2014) and lower buildup of homeostatic sleep pressure (e.g., slow wave power activity: Jenni & Carskadon, 2004; Jenni et al., 2005). Importantly, Crowley et al. (2014) found that adrenal maturity level effects on melatonin added significant information to the shift to later sleep onset on weekdays compared to single effects across chronological age, particularly from pubertal stage three. This points to the relevance of determining age and pubertal status effects separately, as proposed here.

Furthermore, higher pubic hair Tanner ratings have been shown to relate to decreases in sleep duration in girls (but not boys) (Foley et al., 2018), which indicates a more efficient homeostatic recuperative drive ascribed to adrenally induced effects on brain systems that regulate sleep–wake cycles (Campbell, 2011; Greaves et al., 2019). Differently, Jenni et al. (2005) and Jenni and Carskadon (2004) did not show this effect (measured as an exponential decline in slow wave activity across the night) when comparing prepubertal and mature adolescents, but their samples were small.

Together, the results of these few studies suggest that adrenal changes in adolescence may drive delayed bedtime and increased efficiency of sleep pressure dissipation (reduced sleep duration). However, there are insufficient data regarding gonadal maturational effects on adolescent sleep patterns, although there is some evidence that gonadal hormones play a role in circadian entrainment (Hagenauer & Lee, 2012, 2013). How pubertal maturation influences social jetlag across adolescence has not been studied, although it seems to increase with age in this phase of life (Caliandro et al., 2021; Díaz‐Morales & Escribano, 2015; Henderson et al., 2019; Roenneberg et al., 2012; Wittmann et al., 2006).

Nonetheless, the available literature on this issue has many limitations that make it difficult to disentangle age from pubertal effects. These include comparing different pubertal stages irrespective of participants' age (e.g., Carskadon et al., 1993; Sadeh et al., 2009), using low age/pubertal status ranges that do not encompass all of adolescence and pubertal stages (e.g., Borrani et al., 2020; Díaz Morales et al., 2023; Sadeh et al., 2009), grouping PDS scores into artificial categories (Carskadon et al., 1993), excluding late developers for unknown reasons (e.g., Diao et al., 2020) and considering the effects of pubertal *timing* on sleep habits, using pubertal milestones measured prior to the assessment of sleep patterns, which were not of interest here because different pubertal trajectories (tempo) could impact how adolescents sleep over time.

Moreover, the majority of the abovementioned studies assessed adolescents from the developed nations. Whether results apply to other populations is unclear, which is a problem because sleep patterns can be affected by culture (Gariepy et al., 2020; Gradisar et al., 2011; Olds et al., 2010) and early life precursors like low socioeconomic status (SES) (Felden et al., 2015; Olds et al., 2010), both of which also can impact pubertal development (Joos et al., 2018).

The present study cross‐sectionally assessed the sleep habits/preferences reported by 9‐17‐year‐olds of both sexes who were from different socioeconomic backgrounds in Brazil. Because the older an adolescent is, the more pubertally mature he or she is expected to be, not considering age when analysing pubertal status effects on sleep habits can lead to uninterpretable results because more advanced pubertal status may simply reflect changes in sleep due to being older. For instance, older adolescents may be allowed to (or choose to) sleep later at night due to social influences and/or school grade factors (e.g., having more school work or earlier school start times) even if they are less pubertally mature than a younger individual. Teasing apart the effects of age and pubertal status is a complex issue which is either ignored in the literature or addressed by attempting to control for both age and puberty in the same statistical model (França et al., 2025). This latter approach, however, is not adequate because it usually artificially reduces these individual developmental effects. As pointed out by Andrade (2024), when independent variables are intercorrelated (such as age and pubertal status), although their net effect may explain a great portion of variance in the dependent variables (sleep outcomes in the present case), their individual coefficients decrease in proportion to their shared/common variance. Yet, age and pubertal status may show intrinsic differences regarding associations with different sleep patterns. We therefore adopted a statistical approach of independently analysing the influences of the participant's chronological age (in months) and pubertal status considering adrenal and gonadal indicators of pubertal status (Tanner ratings regarding gonadal and adrenal maturation) and self‐rated PDS scores (which measure a mixture of adrenal and gonadal processes) on different sleep outcomes in different statistical models. We then contrasted the results of these models for each sleep outcome to identify which of the developmental indicators explained more variance of each sleep habit. All models were controlled for sex and parental schooling (SES proxy), which can impact both sleep patterns and pubertal status, as mentioned above.

Based on the perfect storm model (see Crowley et al., 2018), we hypothesised that we would replicate that older/more pubertally mature participants would report being more evening oriented and exhibit higher social jetlag (tend to compensate for sleep loss during the week over the weekends) (Caliandro et al., 2021; Díaz‐Morales & Escribano, 2015; Henderson et al., 2019; Roenneberg et al., 2012; Wittmann et al., 2006). We also predicted that sleep patterns at different ages might be better explained by pubertal status than chronological age due to the sexual maturation effects on brain development that could influence sleep physiology (Campbell et al., 2012; Crowley et al., 2018; Foley et al., 2018; Hagenauer & Lee, 2012, 2013; Jenni et al., 2005; Jenni & Carskadon, 2004). Whether adrenal or gonadal maturations would have larger effects, and in which types of sleep habits, was unclear due to the lack of studies on gonadal indicators in the sleep literature. Nonetheless, because adrenal maturation has been more associated with sleep homeostasis than gonadal pubertal status (Campbell et al., 2012; Foley et al., 2018), we expected to find more sleep changes associated with Tanner pubic hair ratings and, possibly, to higher PDS scores (Randler et al., 2009), which also measures some aspects of adrenal development.

## METHOD

### Participants

We used a convenience community sample of 142 consecutive children/adolescents aged 9–17 years who attended a public outpatient clinic for adolescents at a hospital in the city of São Paulo (Hospital São Paulo) during a 4‐month period before the COVID‐19 pandemic. Participants who had a clinical condition that could influence sleep reported by their physicians were excluded. See the Methods section for sample size calculation.

### Procedure

This cross‐sectional study was approved by the Ethical Committee of the Universidade Federal de São Paulo, where the study took place (#64078517.7.0000.5505). Families were approached in the waiting room before the appointments with the doctor in an outpatient clinic that offered free consultations to adolescents. Informed consent from parents and assent from adolescents were obtained from those who agreed to take part. Participation of adolescents involved filling in questionnaires about sleep patterns and preferences and the PDS. The adults responsible for the youngsters provided demographic data and parental years of schooling, used as a proxy for SES (see Farah, 2017). Tanner ratings were determined by highly trained Medical Doctors, who also provided information on clinical conditions that could affect sleep patterns and other health information for exclusion purposes.

### Measures

#### Sleep pattern profiles

Sleep pattern questionnaire: this was a compendium of questions answered by the participants about habitual sleep schedules regarding the time that they went to bed and woke up (as in Gariepy et al., 2020) on weekdays (constrained by rise times imposed by school and other social activities) and weekends (only pertaining to days in which they did not have to rise early to attend church, extra‐curricular activities, etc.). From bed and rise times we estimated time in bed (TIB) during weekdays and weekends, the midpoint of sleep (time of day that coincided with the middle of the sleep period, calculated as the estimated midpoint between the time of going to bed and rise time) during weekdays and weekends, and social jetlag (the difference between the midpoints during weekends and weekdays, measured in hours: Wittmann et al., 2006). For all these calculations, reported minutes of each sleep parameter were transformed into decimals. Bedtimes were reported before and after midnight, so we used the difference from 00:00 in the analyses (negative values indicating time before, and positive ones, after midnight). When calculating social jetlag, we did not correct for individual weekly average sleep need (not sleep debt corrected: see Caliandro et al., 2021) because asking teenagers what they believe was their ideal sleep need made little sense since this is in constant change in this phase of life.

Morningness–Eveningness Scale for Children [MESC; Carskadon et al., 1993 adapted for local use by Finimundi et al., 2012]: this self‐assessment questionnaire is the most popular measure of circadian preferences in paediatric populations (Tonetti et al., 2015) and measures sleep pattern preferences with 10 questions answered on 4 or 5 point scales. Scores are the sum of points (after inverting scores in questions 1, 3, 4, 5, 6, 8 and 10) and range from 10 (maximum eveningness) to 43 (maximum morningness). Based on pilot studies, some minor modifications had to be made to make the scale easier to understand, especially regarding the younger participants (e.g., changing the notation of time of day from 05 h00 to a more familiar format in Brazil: 05:00 h).

#### Developmental status

Age: measured in months between the participants' birthdays and date of assessment.

Pubertal Development Scale [PDS; Carskadon & Acebo, 1993 adapted for local use by Pompeia et al., 2019]: this self‐assessment instrument includes five questions, some of which relate to adrenal and others to gonadal maturation: growth in height, body hair growth and changes in the skin for both sexes, menarche (first menstrual period) and development of the breasts for girls and growth of facial hair and voice changes for boys. Answers are given on 4‐point scales [ranging from not yet started (1 point) to “appeared complete” (4 points)] except for menarche (no = 1 point; yes = 4 points). Scores were the mean number of points.

Tanner staging [Tanner, 1962]: pubertal maturation assessed by highly trained Medical Doctors specialised in Adolescent Medicine which classifies youngsters in one of five stages (1 = prepubertal to 5 = post‐pubertal) of gonadal development [growth of breasts (girls) and genitals (boys)] and adrenal development (pubic hair growth in both sexes). Ratings between two adjacent stages were also permitted (i.e. 4.5 for a rating between stage 4 and 5: Sadeh et al., 2009).

### Statistical analyses

The analyses were undertaken using Statistica v.12 software. The level of significance was *p* ≤ .05. The descriptive analyses include mean (±SD) values by age in years and Tanner ratings. Linear Pearson correlations were determined among age and pubertal measures and among the reported sleep habits and circadian preference (MESC scores), irrespective of developmental measures. Correlations reaching *r* = .30–.50 were regarded as medium effect sizes, whereas those higher than *r* = .50 were considered large effect sizes (Ellis, 2010).

Developmental effects were determined with univariate General Linear Models, including inspection of the acceptability of the pattern of residuals. For each sleep variable (bedtime, rise times and estimated TIB on weekends and weekdays, MESC scores and social jetlag) we ran separate models for each of the developmental factors (age in months, PDS scores, Tanner breast/genitals and Tanner pubic hair ratings), controlling for sex (categorical predictor) and our SES proxy (mean parental schooling as a continuous predictor). All possible interactions of independent variables were included in the models.

For the effects that reached statistical significance we determined partial eta squared (p*η* ^2^) and multiple determination coefficients (*R* ^2^) to indicate the proportion of variance of the dependent measures (sleep variables) explained by independent variables and the models respectively: p*η* ^2^ .6–.13 were medium, and .14 or higher are large effect sizes, whereas *R* ^2^ values of .13–.25 were considered medium effect sizes, and those above .26 are regarded as large effect sizes (Ellis, 2010). Factors and interactions that are not mentioned in the Results section did not reach statistical significance (*p* > .05) and were reported in the Supplementary Materials.

### Sample size calculation

Compared to the present study, prior published findings on the association of pubertal status with sleep measures were mostly based on different types of samples (e.g., of different age/pubertal status ranges) and used distinct experimental designs, measured development and sleep with different metrics, and/or ran statistical models with different sets of predictors (covariates), having shown these associations based on statistically significant effects (*p* values). Additionally, this literature failed to report how sample sizes were determined, did not generally include the effect sizes of results and did not make their datasets available. Hence, we did not have access to data that could be used as a basis to determine the minimum sample size needed to detect these associations in the present experiment. We therefore carried out sample size estimation using the G*Power software (version 3.1.9.7; Heinrich‐Heine‐Universität Düsseldorf, Düsseldorf, Germany: http://www.gpower.hhu.de/) that would yield a sample that was sufficient to detect medium effect sizes (*f* ^2^ = .15, a value provided by this software), which are traditionally regarded as of practical, real‐world utility. Among the models available in G*Power to calculate a priori sample size, the one that is most similar to General Linear Models (proposed here) is “linear multiple regression fixed models, *R* ^2^ deviations from zero”, so this was the selected option. For detecting a medium effect size, with alpha = .05, power = .80 and number of predictors = 7 (developmental measure, sex, parental schooling and all possible interactions), the result retrieved a sample size of 103. We nonetheless tested a higher number of consecutive participants within a four‐month period so that we would have a large enough sample size after possible participant exclusions.

## RESULTS

The dataset can be found at the Open Science Framework depository (https://osf.io/8wvea). Among the 142 consecutively assessed patients who were of the age of interest (9–17 years), 13 were excluded because they presented clinical conditions (reported by their physicians) that could have considerably affected sleep patterns (e.g., severe asthma, sickle cell anaemia, psychiatric disorders). Another six were not included in the analyses because they did not provide any information on their sleep habits, and a further two were removed from the analyses due to difficulties in filling out the questionnaires, even with help. The remaining participants (*n* = 121; 68 girls) had a mean ± SD age of 165 ± 26 months (range: 109–211 months) and their parents had an average of 5.2 ± 2.0 years of schooling (range 0–12 years).

Our measures of interest were self‐reported bed and wake times and sleep schedule preferences and not school shifts because adolescents who do not attend school in the morning can still be woken early and/or even prefer to do so (see de Santana et al., 2025). We had information available regarding school shifts for 91 of our participants. Fifty of them attended school that started in the early morning, 36 in the early afternoon and five in the evening, but the school start time varied in each school shift as often occurs in Brazil.

Descriptive statistics of the analysed sample can be found in Tables 1 and 2 (sleep parameters per age, and pubertal staging per age, respectively). Because the participants were drawn from an outpatient adolescent clinic, some presented conditions that can alter pubertal timing (16 presented precocious puberty, 5 were athletes and many were overweight/obese: see databank for details). These individuals were not excluded because we were interested in associating sleep patterns with stages of puberty irrespective of factors that could anticipate or delay puberty.

**TABLE 1:** Mean (±SD) sleep‐related parameters (in time: hh.mm; or scores) according to age in full years.

Sleep variables	Age in years (*N*)																	
	9 years (3) [mean SD]		10 years (12) [mean SD]		11 years (11) [mean SD]		12 years (23) [mean SD]		13 years (13) [mean SD]		14 years (22) [mean SD]		15 years (11) [mean SD]		16 years (17) [mean SD]		17 years (8) [mean SD]	
Bedtime^a^ Weekend	0.17	1.89	23.45	1.60	0.18	1.76	23.64	1.02	0.31	1.63	0.63	2.12	0.02	1.24	0.88	1.39	0.50	1.34
Rise time^a^ Weekend	11.33	0.58	9.37	1.68	10.14	1.83	9.71	1.87	10.02	2.43	10.35	1.84	9.98	1.80	10.35	2.15	9.72	1.25
Time in bed^a^ Weekend	11.17	1.61	9.88	0.71	9.95	2.30	10.07	1.71	9.71	1.81	9.71	1.51	9.95	1.30	9.53	2.03	9.22	2.19
Bedtime^a^ Weekdays	20.92	1.01	21.85	0.83	22.45	1.40	22.26	1.48	22.38	1.02	23.35	1.85	22.80	1.04	23.01	0.89	22.94	0.94
Rise time^a^ Weekdays	6.00	0.00	6.94	1.21	7.58	1.77	7.34	1.83	8.01	1.73	7.73	1.97	6.17	0.49	6.75	1.71	7.51	2.36
Time in bed^a^ Weekdays	9.08	1.01	9.09	0.64	9.12	2.25	9.08	2.45	9.63	1.27	8.38	2.24	7.37	0.91	7.74	1.84	8.57	2.44
MESC (score)	30.00	2.83	28.08	5.71	27.36	4.52	28.17	7.13	27.77	5.00	25.89	4.84	27.91	3.59	25.47	5.37	27.88	4.52
Social Jetlag^a^	−4.29	0.97	−2.03	1.81	−2.14	1.28	−1.88	1.32	−1.96	1.40	−1.86	1.56	−2.52	1.39	−2.53	1.45	−1.89	1.46

*Note*: MESC = Morningness‐Eveningness Scale for Children scores range from 10 to 43 (smaller values indicate higher eveningness). One participant did not report age reliably so is not included in the table.

^a^ Minutes in decimals.

**TABLE 2:** Distribution of pubertal status (mean ± SD) per sex per age (in years) according to Tanner ratings and Pubertal Development Scale (PDS) scores.

Boys							Girls						
Age (*N*)	Tanner pubic hair [mean SD]		Tanner genitalia [mean SD]		PDS [mean SD]		Age (*N*)	Tanner pubic hair [mean SD]		Tanner breast [mean SD]		PDS [mean SD]	
9 years (0)	‐	‐	‐	‐	‐	‐	9 years (3)	2.00	1.00	1.66	0.57	1.96	0.55
10 years (4)	1.50	1.00	1.25	0.50	1.58	0.41	10 years (8)	3.00	1.19	3.25	1.16	2.37	0.69
11 years (6)	2.08	1.02	2.16	0.98	1.66	0.24	11 years (5)	3.80	1.64	3.50	0.70	2.60	0.58
12 years (12)	3.20	1.58	2.95	1.52	2.03	0.62	12 years (11)	4.81	0.40	4.60	0.69	3.04	0.38
13 years (7)	3.41	1.42	3.58	0.91	2.01	0.50	13 years (6)	4.40	0.89	4.16	0.75	3.06	0.46
14 years (9)	4.44	1.01	4.33	0.82	2.59	0.33	14 years (13)	4.92	0.27	4.69	0.48	3.27	0.42
15 years (7)	4.42	1.13	4.42	1.13	2.54	0.41	15 years (4)	5.00	0.00	5.00	0.00	3.05	0.34
16 years (6)	5.00	0.00	5.00	0.00	2.83	0.34	16 years (11)	4.90	0.30	4.90	0.30	3.52	0.25
17 years (2)	5.00	0.00	5.00	0.00	3.20	0.56	17 years (6)	5.00	0.00	5.00	0.00	3.50	0.48

*Note*: The Tanner scale score varies from 1 to 5 and the PDS from 1 to 4 (higher values indicate higher pubertal status).

Age (in months) correlated significantly, positively and highly (*p* values < .002) with pubertal status measured using the PDS (*r* = .57), the Tanner pubic hair (*r* = .60) and Tanner gonadal ratings (*r* = .65). Detailed results of linear Pearson correlations among sleep‐related variables, irrespective of developmental measures (age and pubertal status), are reported in Table 3. Considering significance determined with Bonferroni correction for multiple comparisons (*p* < .002) and medium to large effect correlations (*r* > .30), we found that, as expected, on both weekends (*r* = −.33) and weekdays (*r* = −.51), participants who slept later spent significantly less TIB, whereas later rise times on weekends and weekdays were associated with more TIB (respectively, *r* = .73; *r* = .62). Individual differences were also observed as there were also positive significant associations between going to bed and rising later on weekends with the same measures on weekdays (respectively *r* = .57; *r* = .30), and TIB on weekends and weekdays (*r* = .34). More evening‐oriented youngsters (with lower MESC scores) also slept (*r* = −.37) and woke up (*r* = −.44) significantly later on weekends and also slept later (*r* = −.35) but did not significantly rise later on weekdays (*r* = −.23) indicating that the MESC scores were in accord with the participants' sleep patterns except when required to rise early on school days. Furthermore, higher values of social jetlag were significantly positively correlated with earlier wake‐up time (*r* = −.43) and less TIB (*r* = −.31) on weekdays, and later bed (*r* = −.55) and rise times (*r* = .62) on weekends, but did not have significantly more TIB on days off (*r* = .18).

**TABLE 3:** Linear correlation between sleep‐related measured parameters, irrespective of developmental stage (age or pubertal status).

	Bedtime weekends	Rise time weekends	TIB weekends	Bedtime weekdays	Rise time weekdays	TIB weekdays	Social jetlag	MESC
Bedtime Weekends	1.00	0.53*	−0.33*	0.57*	0.12	−0.29*	0.55*	−0.37*
Rise time Weekends	0.53*	1.00	0.62*	0.30*	0.30*	0.06	0.62*	−0.44*
Time in bed Weekends	−0.33*	0.62*	1.00	−0.19	0.23	0.34*	0.18	−0.15
Bedtime Weekdays	0.57*	0.30*	−0.19	1.00	0.20	−0.51*	−0.10	−0.35*
Rise time Weekdays	0.12	0.30*	0.23	0.20	1.00	0.73*	−0.43*	−0.23
Time in bed Weekdays	−0.29*	0.06	0.34*	−0.51*	0.73*	1.00	−0.31*	0.04
Social Jetlag	0.55*	0.62*	0.18	−0.10	−0.43*	−0.31*	1.00	−0.14
MESC	−0.37*	−0.44*	−0.15	−0.35*	−0.23	0.04	−0.14	1.00

*Note*: MESC for children (lower scores indicate higher eveningness).

Abbreviations: MESC, Morningness–Eveningness Scale; TIB, time in bed.

* *p* ≤ 0.002 (Bonferroni corrected).

Regarding developmental effects once confounders were accounted for (sex, parental schooling and interactions), a summary of the General Linear Model effects that reached significance can be found in Table 4. Significant results (*p* values ≤ .05) are reported below in detail, including effect sizes. Non‐significant results can be found in the Supplementary Materials.

**TABLE 4:** Summary of explained variance (*R* ^2^) of the tested General Linear Models used to associate sleep‐related variables with developmental measures which reached significant effects [age, Pubertal Development Scale (PDS) scores, Tanner breasts in girls/genitalia in boys (gonadal indicator) and Tanner pubic hair (adrenal indicator) ratings].

Sleep variables	Developmental parameters			
	Age	PDS	Tanner gonadal	Tanner adrenal
Weekends				
Bedtime	‐	‐	‐	0.06
Rise time	‐	‐	‐	‐
Time in bedtime	‐	‐	‐	0.10*
Weekdays				
Bedtime	0.13	‐	‐	0.10
Rise time	‐	‐	‐	‐
Time in bedtime	‐	‐	0.08*	0.07*
MESC score	‐	0.06*	0.08*	‐
Social jetlag	‐	‐	‐	‐

*Note*: ‐ = no significant General Linear Model effects; *were negative associations; *R* ^2^ of 0.13 or above are medium effect sizes. MESC for children (lower scores indicate higher eveningness).

Abbreviation: MESC, Morningness–Eveningness Scale.

There were no significant associations between wakeup times on weekends or weekdays across all four developmental measures (*p* values > .20). In contrast, later bedtimes on weekdays were detected in older participants [*F* (1, 109) = 4.33, *p* < .04; *B* = .04; p*η* ^2^ = .04; model *R* ^2^ = .13; *R*a^2^ = .08] and in those who were more mature in terms of Tanner ratings of pubic hair [*F* (1, 103) = 4.57, *p* < .04; *B* = .75; p*η* ^2^ = .04; model *R* ^2^ = .10; *R*a^2^ = .04]. However, only higher Tanner pubic hair ratings, but not age, were associated with later bedtimes on weekends [*F* (1, 101) = 4.41, *p* < .04; *B* = .87; p*η* ^2^ = .04; *R* ^2^ = .06; *R*a^2^ = −.006].

Models with TIB as the dependent variable showed that more developed adolescents in terms of ratings of Tanner pubic hair [*F* (1,103) = 5.05, *p* < .03; *B* = −.58; p*η* ^2^ = .05; model *R* ^2^ = .07; *R*a^2^ = .0003] and Tanner breasts/genitalia [*F* (1,103) = 4.67, *p* = .03; *B* = −1.05; p*η* ^2^ = .04; model *R* ^2^ = .08; *R*a^2^ = .001] reported significantly less TIB on weekdays, but this was only true on weekends for pubic hair Tanner pubertal status [*F* (1,103) = 6.80, *p* < .02; *B* = −1.09; p*η* ^2^ = .06; model *R* ^2^ = .10; *R*a^2^ = .04]. TIB on weekends was the only tested model in which a variable other than the developmental ones was observed [Tanner pubic hair × parental schooling interaction: *F* (1,103) = 4.50, *p* < .04; *B* = .15; p*η* ^2^ = .001, a minimal effect size; a similar, non‐significant interaction trend was observed for TIB on week days].

As for MESC results, a different pattern of effects was found. Higher eveningness preference (lower MESC scores) was related to higher maturity in respect to breast/genitalia Tanner ratings [*F* (1,105) = 4.43, *p* < .04; *B* = −2.78; p*η* ^2^ = .04; model *R* ^2^ = .08; *R*a^2^ = .022] and PDS scores [*F* (1,105) = 3.85, *p* = .05; *B* = −4.94; p*η* ^2^ = .03; model *R* ^2^ = .06; *R*a^2^ = .0004], but not pubic hair Tanner ratings. Social jetlag was not significantly associated with any of the developmental measures under investigation (*p* values > .20), although the sample as a whole had a misalignment between midpoint sleep on days off and school nights of around 2 h (mean = 2.10, SD = 1.48).

## DISCUSSION

Our results supported our general hypotheses and corroborated findings that during adolescence, both age and pubertal status are associated with changes in sleep patterns due to a combination of psychosocial and physiological factors (Crowley et al., 2018). Furthermore, we found that different aspects of sleep schedules related to distinct developmental markers: age only influences bedtimes on school nights, suggesting that age reflects a societal factor (such as parents accepting that older adolescents can sleep later), whereas higher adrenal maturity (pubic hair Tanner stages) is related to later bedtime (associated with a decrease in buildup of sleep pressure/phase delay: Crowley et al., 2014) and to less sleep need (TIB) on weekdays and days off, which may indicate physiological changes associated with increased efficiency of sleep pressure dissipation (Campbell et al., 2012). In contrast, increased eveningness preference, measured with MESC scores, was only associated with gonadal pubertal status (genital/breast Tanner rating) and PDS scores (possibly due to the effects of the PDS questions that related to the latter neuroendocrine changes). Social jetlag, however, did not differ across all tested developmental markers. Next, we contrast our results with the literature in more detail.

Unsurprisingly, the time of day in which the sample woke on weekdays was not related to any of the developmental measures because this is usually determined by fixed school start times and/or family routines. Differently, older age was found to associate with later bedtime on weekdays, our only finding that reached a medium effect size, suggesting that sleep schedules are somewhat dependent on social determinants (e.g., higher use of electronic equipment, higher academic burden and/or independence from parentally set bedtimes) (Chen et al., 2024), which might explain why it is commonly reported that older adolescents tend to be more evening oriented (Carskadon et al., 1993; Díaz‐Morales & Escribano, 2015; Roenneberg et al., 2004; Thorleifsdottir et al., 2002). It should nevertheless be considered that later bedtimes do not solely indicate social factors, but also physiological changes in circadian and homeostatic traits that have been related to pubertal maturation (Crowley et al., 2014).

Regarding later bedtimes on weekdays and days off in more adrenally mature individuals, our results in Brazilian adolescents were consistent with data obtained in samples from other countries [e.g., Crowley et al., 2014; Foley et al., 2018; Jenni & Carskadon, 2004; Jenni et al., 2005; and possibly by Randler et al., 2009 regarding PDS questions related to adrenal changes], later dim light melatonin onset (Crowley et al., 2014) and phase of melatonin secretion (Carskadon et al., 1997, 2004), which are markers of delayed circadian phase, and/or of lower buildup of homeostatic sleep pressure. Notwithstanding, our data indicate that later bedtimes may be more sensitive markers of homeostatic factors than circadian preferences. The reasons for this are twofold. Firstly, MESC scores did not associate with age (as found by de Santana et al., 2025) and adrenal markers of puberty, yet higher eveningness preferences were related to more advanced gonadal pubertal status (Tanner breast/genitalia ratings and PDS scores, a scale that includes questions regarding gonadal maturation that could have driven these effects). This confirms prior work that suggests that gonadal hormones play a role in circadian entrainment (Hagenauer & Lee, 2012), although this must be confirmed because very few studies specifically considered gonadal pubertal status markers. Hence, delayed sleep timing might be more sensitive to pubertal maturity (particularly adrenal) than age, even though adrenal and gonadal maturational effects are difficult to disentangle, especially considering differential individual variations in pubertal timing and pubertal trajectories (Dorn et al., 2006, 2019). The same is true regarding challenges in the differentiation of homeostatic and circadian factors on sleep patterns (see Panjeh et al., 2022).

The second reason we believed adrenal maturation related more to homeostatic sleep processes than circadian phase delay is our finding regarding sleep duration. Descriptively speaking, our TIB results were compatible with data from adolescents from many different countries (e.g., Gariepy et al., 2020), but a decrease across ages, found in other samples (e.g., Olds et al., 2010), was not observed here. Because these age effects have generally been shown to be quite small (minus 14 min on weekdays and minus 7 min on weekends per year: Olds et al., 2010), it cannot be excluded that our sample was not large enough to pick up this trend. Nonetheless, higher Tanner ratings of pubic hair pubertal status associated with less TIB on weekdays (similarly to Tanner for breast/genitalia ratings), and also on weekends, indicating less sleep need (possibly more mature patterns of dissipation of homeostatic sleep pressure) (Crowley et al., 2018; Hagenauer & Lee, 2013), a finding that was not observed concerning age effects and those of gonadal maturation. Similar results were reported in adolescents with higher PDS scores (e.g., Diao et al., 2020; Mozafarian et al., 2023; Randler et al., 2009), a scale which includes items that assess adrenal pubertal status.

Hence, our findings corroborate those of Foley et al. (2018), who studied pubertal timing and tempo longitudinally and showed that sleep–wake behaviours relate more to pubic hair maturity than gonadal maturation, although this was evident only in girls and not boys, which could have been due to their earlier waves of assessment having involved 8‐ to 11‐year‐olds, an age in which puberty has not yet started in most boys (Dorn et al., 2006, 2019; Joos et al., 2018). Additionally, adrenal endocrine development may be more important in determining the maturation of homeostatic processes (Campbell et al., 2012; Crowley et al., 2018), possibly resulting from adrenally induced effects on brain systems that regulate sleep–wake cycles (Campbell, 2011; Greaves et al., 2019). Yet, this does not corroborate data from some other studies in terms of physiological and behavioural measures [e.g., Jenni & Carskadon, 2004 and Jenni et al., 2005, who used small samples; Kirshenbaum et al., 2023, who assessed subjective sleep duration during a short period], calling for more studies in this respect.

Regarding circadian irregularity between sleep time on weekends and days off, our sample as a whole reported a social jetlag of around 2 h as others before (Caliandro et al., 2021; Díaz‐Morales & Escribano, 2015; Henderson et al., 2019; Roenneberg et al., 2012; Wittmann et al., 2006), a misalignment associated with negative health outcomes and probably worse quality of life, although this varies across different sociocultural groups (e.g., Caliandro et al., 2021). However, we did not show the predicted increase in social jetlag associated with any of our measures of development, even though some publications found it to increase with age (e.g., Díaz‐Morales & Escribano, 2015; Roenneberg et al., 2012). Our sample was possibly not large enough to detect this effect because the effect sizes of these age‐related changes, when reported, are quite small (e.g., Díaz‐Morales & Escribano, 2015), suggesting they are probably of low clinical or practical relevance. We found no study that specifically showed associations of social jetlag with pubertal maturation.

SES measured as parental schooling was considerably varied in our sample, as expected in a non‐developed country, although it had very subtle effects, only having reached significance in a single model with a minute effect size (Tanner pubic hair interacted with parental schooling only for TIB on weekends), which can be ascribed to SES‐related effects on both sleep (Felden et al., 2015; Olds et al., 2010) and pubertal maturation (Joos et al., 2018). Note that prior SES effects on adolescent sleep have been generally observed using very large samples (for a review, see Felden et al., 2015), so we may have lacked power to detect this association in the other models.

In sum, age seems to associate with later bedtimes on school nights due to societal and not pubertal aspects, while adrenal changes were the most sensitive to sleep pattern changes, albeit modest ones, having been more consistently associated with later bedtimes (suggesting lower buildup of sleep pressure and/or circadian phase delay) and less TIB (indicative of increases in the efficiency in dissipating sleep pressure across the night), which might be protective compared to less adrenally mature and/or age‐matched peers if adolescents are not allowed to sleep as much as they need to. Differently, gonadal changes, which develop independently from adrenal maturity (Dorn et al., 2006, 2019; Joos et al., 2018) were better related to a delayed circadian/homeostatic indicator (MESC scores).

Even though our study was cross‐sectional, involved a limited sample and our statistical models only explained a small portion of the variance in sleep/circadian variables, the results point to the need to better explore pubertal associations with sleep and also other variables that better explain adolescents' sleep–wake pattern preferences. If our findings are confirmed, they would suggest that adolescents with higher adrenal puberty status can sleep later and have a lower sleep need than less mature age/sex‐matched peers because their sleep patterns are more akin to those of adults, although lower sleep duration in more sexually mature individuals has been regarded as possibly resulting from higher stress caused by physical and/or psychological changes brought about by puberty (e.g., Diao et al., 2020). A more adrenally mature sleep pattern could be protective in modern society, in which adolescents do not have the opportunity to sleep as much as they need to (see Chen et al., 2024). In contrast, being only older or only more gonadally mature than peers, could actually make adolescents more prone to sleep later while still needing more sleep, increasing their vulnerability to the negative effects of sleep deprivation on academic performance, physical and mental health (e.g., Crowley et al., 2018; Gariepy et al., 2020; Henderson et al., 2019).

Our study is mainly limited by the sample size which, despite being sufficient to pick up developmental alterations, was not large enough to conduct separate analyses by sex, which we nonetheless controlled for sex. We also did not assess sleep quality (which may associate more highly with pubertal status: Kirshenbaum et al., 2023) nor sleep patterns with objective measures (see Gariepy et al., 2020; Kirshenbaum et al., 2023), although the changes in sleep patterns across age generally complied with other international studies that used actigraphy in adolescents (Gradisar et al., 2011). We also included in our community sample some individuals with conditions that can alter pubertal timing/tempo because our aim was to associate pubertal status with sleep profiles, not to determine factors that affect either sleep or puberty. On the upside, we used a sample spanning the whole phase of life in which puberty occurs, employed the gold‐standard of pubertal maturation assessment (Tanner ratings by experienced clinicians) and did so in a diverse sample of Brazilian adolescents, including some with very low SES, which is underrepresented in publications in this field. It remains to be determined whether our results hold for adolescents from different sociocultural and economic scenarios (see Gariepy et al., 2020; Gradisar et al., 2011; Olds et al., 2010), individuals with no clinical problems and the extent to which individual differences and specific pathologies differently influence the associations of gonadal and adrenal maturation with sleep patterns.

Finally, we reached our conclusions by comparing the explained variance of sleep habits in separate models for age and pubertal maturity markers, which is not ideal. Identifying the effects of pubertal status that are not confounded by being older is a complex problem (França et al., 2025), which is not solved by controlling for age and pubertal status in the same statistical models because they intercorrelate [although not near perfectly as is generally found (see França et al., 2025)], leading to reductions of their individual effects in proportion to their shared/common variance (Andrade, 2024). A possible way to avoid this limitation is to use a longitudinal design in which participants' pubertal status is evaluated over time at exactly the same ages (in months of age and not years, since pubertal status can advance across 1 year) (França et al., 2025). This allows the control of the effects of age without artificially reducing pubertal effects. This way, it should be possible to investigate to what extent pubertal development itself, as opposed to non‐pubertally induced physiological and psychosocial changes, affects sleep habits/preferences. Our cross‐sectional results, albeit with a limited sample size, point to a possible differential role of adrenal and gonadal pubertal maturity on different sleep habit parameters, which should be further investigated using this particular type of longitudinal design, preferably employing mixed effect models that account for fixed and random effects to account for individual differences (because our fixed effect sizes were small). Longitudinal upcoming research should also include specific measurements of indicators of reproductive and somatotropic axes, which have interacting effects on sleep at this age (see Lucien et al., 2021), in addition to sociocultural factors that can affect sleep schedules (e.g., family relations: Díaz Morales et al., 2023).

## CONCLUSION

Age, adrenal and gonadal pubertal maturity status measures associate differently with sleep patterns and circadian preferences during adolescence. Older age only related to later bedtimes on weekdays, suggesting this is more socially than physiologically determined, whereas circadian phase delay (MESC scores) only associated with gonadal maturation. Differently, we found indications that adrenal maturation may be a more sensitive marker of homeostatic changes (lower buildup of sleep pressure indicated by later bedtimes, and/or a more efficient sleep pressure dissipation, indicated by lower sleep need).

## AUTHOR CONTRIBUTIONS

**Yessica Alejandra Martínez‐Sánchez:** Writing – original draft; formal analysis; data curation. **Debora Cristina Hipolide:** Writing – review and editing; supervision; visualization. **Rafaella Sales de Freitas:** Writing – review and editing; methodology; formal analysis; investigation; data curation. **Sabine Pompeia:** Conceptualization; funding acquisition; writing – review and editing; methodology; formal analysis; project administration; supervision; visualization.

## FUNDING INFORMATION

This work was supported by Fundação de Amparo à Pesquisa do Estado de São Paulo (FAPESP; #2017/01908‐7), Coordenação de Aperfeiçoamento de Pessoal de Nível Superior (CAPES; finance code 001), Conselho Nacional de Desenvolvimento Científico e Tecnológico (CNPq), and Associação Fundo de Incentivo à Pesquisa (AFIP), all non‐profit organisations that sponsor research in Brazil.

## CONFLICT OF INTEREST STATEMENT

The authors declare no conflicts of interest.

## Supporting information


Data S1:

## Data Availability

The databank can be found at the OSF depository (https://osf.io/8wvea).
