# Supplementary material for: Changes in sleep patterns in adolescents are more associated with pubertal indicators than age: A perfect storm with a dash of hormones
Source: Br J Dev Psychol. 2025 Nov 18;44(2):437–53. doi: 10.1111/bjdp.70028 (PMC13155287; doi:10.1111/bjdp.70028)
Supplement: Supplementary file 1 — Data S1: [file BJDP-44-437-s001.docx]

**SUPPLEMENTARY MATERIAL**

Find below the results of the univariate general linear models, including non-significant effects, per sleep measure (dependent variables) considering different developmental predictors (age in months, Pubertal Development Scale scores, Tannar pubic hair and breast/genitalia ratings), controlling for sex and mean parental number of years of schooling plus all possible interactions (indicates by *). Values in bold reached statistical significance and are detailed in the main document, including their effect sizes.

Table 1S. Results of univariate general linear models regarding self-reported bed times on weekdays.

|  | Effects | \| SS \| \| --- \| | \| DF Freedom \| \| --- \| | \| MS \| \| --- \| | \| F \| \| --- \| | \| p \| \| --- \| |
| --- | --- | --- | --- | --- | --- | --- | --- | --- | --- | --- | --- |
| Age in in months (SE±1.324) | \| Intercept \| \| --- \| | 11.6986 | 1 | 11.69862 | 6.671365 | 0.011122 |
|  | \| sex \| \| --- \| | 2.7189 | 1 | 2.71890 | 1.550508 | 0.215731 |
|  | \| **age in months** \| \| --- \| | **7.5975** | **1** | **7.59747** | **4.332604** | **0.039731** |
|  | \| mean parental schooling \| \| --- \| | 2.0239 | 1 | 2.02393 | 1.154186 | 0.285047 |
|  | \| sex*age in months \| \| --- \| | 3.0006 | 1 | 3.00056 | 1.711126 | 0.193591 |
|  | \| sex*mean parental schooling \| \| --- \| | 2.7180 | 1 | 2.71795 | 1.549965 | 0.215811 |
|  | \| age in months*mean parental schooling \| \| --- \| | 1.6972 | 1 | 1.69722 | 0.967872 | 0.327390 |
|  | \| sex*age in months*mean parental schooling \| \| --- \| | 2.8100 | 1 | 2.81000 | 1.602457 | 0.208254 |
|  | \| Error \| \| --- \| | 191.1377 | 109 | 1.75356 |  |  |
|  |  |  |  |  |  |  |
| PDS (SE±1.327) | \| Intercept \| \| --- \| | 12.6963 | 1 | 12.69626 | 7.206149 | 0.008400 |
|  | \| sex \| \| --- \| | 0.0749 | 1 | 0.07485 | 0.042485 | 0.837083 |
|  | \| mean parental schooling \| \| --- \| | 1.0522 | 1 | 1.05216 | 0.597182 | 0.441328 |
|  | \| **PDS score** \| \| --- \| | **6.5105** | **1** | **6.51049** | **3.695225** | **0.057179** |
|  | \| sex*mean parental schooling \| \| --- \| | 0.5625 | 1 | 0.56251 | 0.319268 | 0.573209 |
|  | \| sex*PDS score \| \| --- \| | 0.4102 | 1 | 0.41022 | 0.232833 | 0.630399 |
|  | \| mean parental schooling*PDS score \| \| --- \| | 0.9728 | 1 | 0.97283 | 0.552157 | 0.459036 |
|  | \| sex*mean parental schooling*PDS score \| \| --- \| | 0.6488 | 1 | 0.64884 | 0.368272 | 0.545210 |
|  | \| Error \| \| --- \| | 192.0433 | 109 | 1.76187 |  |  |
|  |  |  |  |  |  |  |
| Tanner pubic (SE±1.375) | \| Intercept \| \| --- \| | 17.0531 | 1 | 17.05315 | 9.017461 | 0.003356 |
|  | \| sex \| \| --- \| | 1.1135 | 1 | 1.11353 | 0.588821 | 0.444631 |
|  | \| mean parental schooling \| \| --- \| | 3.5123 | 1 | 3.51231 | 1.857257 | 0.175914 |
|  | \| **Tanner pubic hair** \| \| --- \| | **8.6476** | **1** | **8.64762** | **4.572738** | **0.034849** |
|  | \| sex*mean parental schooling \| \| --- \| | 2.7997 | 1 | 2.79967 | 1.480425 | 0.226489 |
|  | \| sex*Tanner pubic hair \| \| --- \| | 1.8948 | 1 | 1.89476 | 1.001923 | 0.319191 |
|  | \| mean parental schooling*Tanner pubic hair \| \| --- \| | 3.5928 | 1 | 3.59278 | 1.899810 | 0.171086 |
|  | \| sex*mean parental schooling*Tanner pubic hair \| \| --- \| | 3.2808 | 1 | 3.28080 | 1.734841 | 0.190717 |
|  | \| Error \| \| --- \| | 194.7859 | 103 | 1.89113 |  |  |
|  |  |  |  |  |  |  |
| Tanner bre/gen (SE± 1.326) | \| Intercept \| \| --- \| | 14.4725 | 1 | 14.47246 | 8.231058 | 0.004997 |
|  | \| sex \| \| --- \| | 0.0000 | 1 | 0.00000 | 0.000000 | 1.000000 |
|  | \| mean parental schooling \| \| --- \| | 1.0411 | 1 | 1.04110 | 0.592114 | 0.443364 |
|  | \| Tanner breast/genitalia \| \| --- \| | 5.8176 | 1 | 5.81756 | 3.308676 | 0.071820 |
|  | \| sex*mean parental schooling \| \| --- \| | 0.5677 | 1 | 0.56774 | 0.322898 | 0.571108 |
|  | \| sex*Tanner breast/genitalia \| \| --- \| | 0.0930 | 1 | 0.09302 | 0.052903 | 0.818542 |
|  | \| mean parental schooling*Tanner breast/genitalia \| \| --- \| | 0.6746 | 1 | 0.67458 | 0.383662 | 0.537018 |
|  | \| sex*mean parental schooling*Tanner bre/gen \| \| --- \| | 0.7332 | 1 | 0.73318 | 0.416986 | 0.519881 |
|  | \| Error \| \| --- \| | 181.1023 | 103 | 1.75827 |  |  |

N.B. SS=sum of squares; DF=degrees of freedom; MS=mean sum of squares; F=F values; p=p values; SE=standard error of estimates (using sigma-restricted parameterization; effective hypothesis decomposition); PDS=Pubertal Development Scale scores (self-assessed); Tanner pubic and bre/gen=Tanner Pubic hair (both sexes) and breast (girls) and genital (boys) ratings by experienced clinicians, respectively; *=interactions; values in bold=p≤.05.

Table 2S. Results of univariate general linear models regarding self-reported wake up times on weekdays.

|  | Effects | \| SS \| \| --- \| | \| DF Freedom \| \| --- \| | \| MS \| \| --- \| | \| F \| \| --- \| | \| p \| \| --- \| |
| --- | --- | --- | --- | --- | --- | --- | --- | --- | --- | --- | --- |
| Age in in months (SE±1.781) | \| Intercept \| \| --- \| | 14.0821 | 1 | 14.08207 | 4.440344 | 0.037393 |
|  | \| sex \|  \| \| --- \| --- \| | 2.7305 | 1 | 2.73047 | 0.860970 | 0.355518 |
|  | \| age in months \| \| --- \| | 0.0124 | 1 | 0.01238 | 0.003902 | 0.950304 |
|  | \| mean parental schooling \| \| --- \| | 0.0754 | 1 | 0.07539 | 0.023771 | 0.877754 |
|  | \| sex*age in months \| \| --- \| | 2.2661 | 1 | 2.26613 | 0.714554 | 0.399788 |
|  | \| sex*mean parental schooling \| \| --- \| | 2.1732 | 1 | 2.17317 | 0.685243 | 0.409596 |
|  | \| age in months*mean parental schooling \| \| --- \| | 0.0093 | 1 | 0.00929 | 0.002930 | 0.956929 |
|  | \| sex*age in months*mean parental schooling \| \| --- \| | 1.7802 | 1 | 1.78022 | 0.561338 | 0.455336 |
|  | \| Error \| \| --- \| | 345.6817 | 109 | 3.17139 |  |  |
|  |  |  |  |  |  |  |
| PDS (SE±1.769) | \| Intercept \| \| --- \| | 41.0598 | 1 | 41.05978 | 13.12031 | 0.000445 |
|  | \| sex \| \| --- \| | 1.6377 | 1 | 1.63765 | 0.52330 | 0.470987 |
|  | \| mean parental schooling \| \| --- \| | 1.3087 | 1 | 1.30865 | 0.41817 | 0.519212 |
|  | \| PDS score \| \| --- \| | 0.6350 | 1 | 0.63502 | 0.20292 | 0.653272 |
|  | \| sex*mean parental schooling \| \| --- \| | 0.1868 | 1 | 0.18675 | 0.05967 | 0.807470 |
|  | \| sex*PDS score \| \| --- \| | 0.9818 | 1 | 0.98182 | 0.31373 | 0.576547 |
|  | \| mean parental schooling*PDS score \| \| --- \| | 0.7784 | 1 | 0.77835 | 0.24872 | 0.618985 |
|  | \| sex*mean parental schooling*PDS score \| \| --- \| | 0.0132 | 1 | 0.01315 | 0.00420 | 0.948426 |
|  | \| Error \| \| --- \| | 341.1134 | 109 | 3.12948 |  |  |
|  |  |  |  |  |  |  |
| Tanner pubic (SE±1.745) | \| Intercept \| \| --- \| | 66.7068 | 1 | 66.70677 | 21.91578 | 0.000009 |
|  | \| sex \| \| --- \| | 2.0185 | 1 | 2.01849 | 0.66315 | 0.417328 |
|  | \| mean parental schooling \| \| --- \| | 3.8412 | 1 | 3.84118 | 1.26198 | 0.263889 |
|  | \| Tanner pubic hair \| \| --- \| | 2.4318 | 1 | 2.43185 | 0.79896 | 0.373489 |
|  | \| sex*mean parental schooling \| \| --- \| | 0.8110 | 1 | 0.81099 | 0.26644 | 0.606836 |
|  | \| sex*Tanner pubic hair \| \| --- \| | 0.8640 | 1 | 0.86395 | 0.28384 | 0.595342 |
|  | \| mean parental schooling*Tanner pubic hair \| \| --- \| | 3.4800 | 1 | 3.47998 | 1.14331 | 0.287454 |
|  | \| sex*mean parental schooling*Tanner pubic hair \| \| --- \| | 0.1433 | 1 | 0.14326 | 0.04707 | 0.828675 |
|  | \| Error \| \| --- \| | 313.5091 | 103 | 3.04378 |  |  |
|  |  |  |  |  |  |  |
| Tanner bre/gen (SE± 1.723) | \| Intercept \| \| --- \| | 73.4172 | 1 | 73.41718 | 24.72340 | 0.000003 |
|  | \| sex \| \| --- \| | 4.1585 | 1 | 4.15854 | 1.40040 | 0.239381 |
|  | \| mean parental schooling \| \| --- \| | 4.6086 | 1 | 4.60858 | 1.55195 | 0.215674 |
|  | \| Tanner breast/genitalia \| \| --- \| | 3.0220 | 1 | 3.02200 | 1.01767 | 0.315437 |
|  | \| sex*mean parental schooling \| \| --- \| | 2.2671 | 1 | 2.26708 | 0.76344 | 0.384285 |
|  | \| sex*Tanner breast/genitalia \| \| --- \| | 2.2874 | 1 | 2.28739 | 0.77028 | 0.382172 |
|  | \| mean parental schooling*Tanner breast/genitalia \| \| --- \| | 4.2704 | 1 | 4.27041 | 1.43807 | 0.233203 |
|  | \| sex*mean parental schooling*Tanner bre/gen \| \| --- \| | 0.9733 | 1 | 0.97325 | 0.32774 | 0.568238 |
|  | \| Error \| \| --- \| | 305.8628 | 103 | 2.96954 |  |  |

N.B. SS=sum of squares; DF=degrees of freedom; MS=mean sum of squares; F=F values; p=p values; SE=standard error of estimates (using sigma-restricted parameterization; effective hypothesis decomposition); PDS=Pubertal Development Scale scores (self-assessed); Tanner pubic and bre/gen=Tanner Pubic hair (both sexes) and breast (girls) and genital (boys) ratings by experienced clinicians, respectively; *=interactions.

Table 3S. Results of univariate general linear models regarding estimated time in bed (TIB) on weekdays.

|  | Effects | \| SS \| \| --- \| | \| DF Freedom \| \| --- \| | \| MS \| \| --- \| | \| F \| \| --- \| | \| p \| \| --- \| |
| --- | --- | --- | --- | --- | --- | --- | --- | --- | --- | --- | --- |
| Age in in months (SE±1.950) | \| Intercept \| \| --- \| | 51.4510 | 1 | 51.45096 | 13.53386 | 0.000366 |
|  | \| sex \|  \| \| --- \| --- \| | 10.8987 | 1 | 10.89874 | 2.86685 | 0.093278 |
|  | \| age in months \| \| --- \| | 2.3528 | 1 | 2.35284 | 0.61890 | 0.433164 |
|  | \| mean parental schooling \| \| --- \| | 9.1865 | 1 | 9.18646 | 2.41644 | 0.122966 |
|  | \| sex*age in months \| \| --- \| | 9.9476 | 1 | 9.94764 | 2.61667 | 0.108637 |
|  | \| sex*mean parental schooling \| \| --- \| | 10.2809 | 1 | 10.28088 | 2.70432 | 0.102958 |
|  | \| age in months*mean parental schooling \| \| --- \| | 1.4553 | 1 | 1.45533 | 0.38282 | 0.537391 |
|  | \| sex*age in months*mean parental schooling \| \| --- \| | 9.0634 | 1 | 9.06344 | 2.38408 | 0.125475 |
|  | \| Error \| \| --- \| | 414.3797 | 109 | 3.80165 |  |  |
|  |  |  |  |  |  |  |
| PDS (SE±1.977) | \| Intercept \| \| --- \| | 99.4203 | 1 | 99.42028 | 25.42954 | 0.000002 |
|  | \| sex \| \| --- \| | 2.4127 | 1 | 2.41274 | 0.61713 | 0.433822 |
|  | \| mean parental schooling \| \| --- \| | 4.7076 | 1 | 4.70764 | 1.20411 | 0.274920 |
|  | \| PDS score \| \| --- \| | 11.2121 | 1 | 11.21211 | 2.86781 | 0.093224 |
|  | \| sex*mean parental schooling \| \| --- \| | 1.3975 | 1 | 1.39748 | 0.35744 | 0.551169 |
|  | \| sex*PDS score \| \| --- \| | 2.6613 | 1 | 2.66132 | 0.68071 | 0.411145 |
|  | \| mean parental schooling*PDS score \| \| --- \| | 3.4915 | 1 | 3.49153 | 0.89306 | 0.346740 |
|  | \| sex*mean parental schooling*PDS score \| \| --- \| | 0.8468 | 1 | 0.84677 | 0.21658 | 0.642583 |
|  | \| Error \| \| --- \| | 426.1505 | 109 | 3.90964 |  |  |
|  |  |  |  |  |  |  |
| Tanner pubic (SE±2.003) | \| Intercept \| \| --- \| | 151.2154 | 1 | 151.2154 | 37.70218 | 0.000000 |
|  | \| sex \| \| --- \| | 6.1305 | 1 | 6.1305 | 1.52849 | 0.219150 |
|  | \| mean parental schooling \| \| --- \| | 14.6996 | 1 | 14.6996 | 3.66502 | 0.058341 |
|  | \| **Tanner pubic hair** \| \| --- \| | **20.2511** | **1** | **20.2511** | **5.04916** | **0.026771** |
|  | \| sex*mean parental schooling \| \| --- \| | 6.6243 | 1 | 6.6243 | 1.65162 | 0.201621 |
|  | \| sex*Tanner pubic hair \| \| --- \| | 5.3176 | 1 | 5.3176 | 1.32583 | 0.252215 |
|  | \| mean parental schooling*Tanner pubic hair \| \| --- \| | 14.1446 | 1 | 14.1446 | 3.52664 | 0.063219 |
|  | \| sex*mean parental schooling*Tanner pubic hair \| \| --- \| | 4.7952 | 1 | 4.7952 | 1.19558 | 0.276757 |
|  | \| Error \| \| --- \| |  |  |  |  |  |
|  |  |  |  |  |  |  |
| Tanner bre/gen (SE± 1.921) | \| Intercept \| \| --- \| | 153.0825 | 1 | 153.0825 | 41.48108 | 0.000000 |
|  | \| sex \| \| --- \| | 4.1591 | 1 | 4.1591 | 1.12699 | 0.290901 |
|  | \| mean parental schooling \| \| --- \| | 10.0305 | 1 | 10.0305 | 2.71799 | 0.102270 |
|  | \| **Tanner breast/genitalia** \| \| --- \| | **17.2254** | **1** | **17.2254** | **4.66761** | **0.033055** |
|  | \| sex*mean parental schooling \| \| --- \| | 5.1039 | 1 | 5.1039 | 1.38300 | 0.242302 |
|  | \| sex*Tanner breast/genitalia \| \| --- \| | 3.3029 | 1 | 3.3029 | 0.89501 | 0.346338 |
|  | \| mean parental schooling*Tanner breast/genitalia \| \| --- \| | 8.3395 | 1 | 8.3395 | 2.25978 | 0.135833 |
|  | \| sex*mean parental schooling*Tanner bre/gen \| \| --- \| | 3.3959 | 1 | 3.3959 | 0.92019 | 0.339672 |
|  | \| Error \| \| --- \| | 380.1130 | 103 | 3.6904 |  |  |

N.B. SS=sum of squares; DF=degrees of freedom; MS=mean sum of squares; F=F values; p=p values; SE=standard error of estimates (using sigma-restricted parameterization; effective hypothesis decomposition); PDS=Pubertal Development Scale scores (self-assessed); Tanner pubic and bre/gen=Tanner Pubic hair (both sexes) and breast (girls) and genital (boys) ratings by experienced clinicians, respectively; *=interactions; values in bold=p≤.05.

Table 4S. Results of univariate general linear models regarding self-reported bed times on weekends.

|  | Effects | \| SS \| \| --- \| | \| DF Freedom \| \| --- \| | \| MS \| \| --- \| | \| F \| \| --- \| | \| p \| \| --- \| |
| --- | --- | --- | --- | --- | --- | --- | --- | --- | --- | --- | --- |
| Age in in months (SE±1.950) | \| Intercept \| \| --- \| | 1.9500 | 1 | 1.949980 | 0.792094 | 0.375464 |
|  | \| sex \|  \| \| --- \| --- \| | 2.9361 | 1 | 2.936065 | 1.192648 | 0.277249 |
|  | \| age in months \| \| --- \| | 0.0350 | 1 | 0.034990 | 0.014213 | 0.905325 |
|  | \| mean parental schooling \| \| --- \| | 2.0351 | 1 | 2.035119 | 0.826678 | 0.365278 |
|  | \| sex*age in months \| \| --- \| | 1.5244 | 1 | 1.524375 | 0.619211 | 0.433080 |
|  | \| sex*mean parental schooling \| \| --- \| | 2.6685 | 1 | 2.668534 | 1.083975 | 0.300157 |
|  | \| age in months*mean parental schooling \| \| --- \| | 0.0042 | 1 | 0.004170 | 0.001694 | 0.967248 |
|  | \| sex*age in months*mean parental schooling \| \| --- \| | 1.2515 | 1 | 1.251547 | 0.508386 | 0.477390 |
|  | \| Error \| \| --- \| | 263.4130 | 107 | 2.461803 |  |  |
|  |  |  |  |  |  |  |
| PDS (SE±1.977) | \| Intercept \| \| --- \| | 2.3050 | 1 | 2.304996 | 0.912858 | 0.341511 |
|  | \| sex \| \| --- \| | 2.1857 | 1 | 2.185702 | 0.865613 | 0.354267 |
|  | \| mean parental schooling \| \| --- \| | 0.5523 | 1 | 0.552275 | 0.218720 | 0.640968 |
|  | \| PDS score \| \| --- \| | 3.1287 | 1 | 3.128706 | 1.239075 | 0.268144 |
|  | \| sex*mean parental schooling \| \| --- \| | 1.3256 | 1 | 1.325591 | 0.524980 | 0.470307 |
|  | \| sex*PDS score \| \| --- \| | 2.1895 | 1 | 2.189539 | 0.867133 | 0.353846 |
|  | \| mean parental schooling*PDS score \| \| --- \| | 0.5052 | 1 | 0.505188 | 0.200072 | 0.655567 |
|  | \| sex*mean parental schooling*PDS score \| \| --- \| | 0.8619 | 1 | 0.861926 | 0.341353 | 0.560279 |
|  | \| Error \| \| --- \| | 270.1785 | 107 | 2.525033 |  |  |
|  |  |  |  |  |  |  |
| Tanner pubic (SE±2.003) | \| Intercept \| \| --- \| | 9.7574 | 1 | 9.75742 | 3.714579 | 0.056749 |
|  | \| sex \| \| --- \| | 4.0945 | 1 | 4.09447 | 1.558735 | 0.214737 |
|  | \| mean parental schooling \| \| --- \| | 7.5908 | 1 | 7.59078 | 2.889752 | 0.092222 |
|  | \| Tanner pubic hair \| \| --- \| | 11.5795 | 1 | 11.57952 | 4.408237 | 0.038258 |
|  | \| sex*mean parental schooling \| \| --- \| | 3.8870 | 1 | 3.88701 | 1.479757 | 0.226648 |
|  | \| sex*Tanner pubic hair \| \| --- \| | 4.0553 | 1 | 4.05527 | 1.543811 | 0.216928 |
|  | \| mean parental schooling*Tanner pubic hair \| \| --- \| | 7.7764 | 1 | 7.77642 | 2.960424 | 0.088388 |
|  | \| sex*mean parental schooling*Tanner pubic hair \| \| --- \| | 3.3356 | 1 | 3.33560 | 1.269838 | 0.262467 |
|  | \| Error \| \| --- \| | 265.3060 | 101 | 2.62679 |  |  |
|  |  |  |  |  |  |  |
| Tanner bre/gen (SE± 1.921) | \| Intercept \| \| --- \| | 4.8161 | 1 | 4.816122 | 1.851840 | 0.176598 |
|  | \| sex \| \| --- \| | 0.7651 | 1 | 0.765119 | 0.294195 | 0.588740 |
|  | \| mean parental schooling \| \| --- \| | 2.8867 | 1 | 2.886738 | 1.109976 | 0.294601 |
|  | \| Tanner breast/genitalia \| \| --- \| | 5.6915 | 1 | 5.691482 | 2.188424 | 0.142164 |
|  | \| sex*mean parental schooling \| \| --- \| | 0.2600 | 1 | 0.260020 | 0.099980 | 0.752506 |
|  | \| sex*Tanner breast/genitalia \| \| --- \| | 0.5013 | 1 | 0.501325 | 0.192764 | 0.661564 |
|  | \| mean parental schooling*Tanner breast/genitalia \| \| --- \| | 2.4421 | 1 | 2.442127 | 0.939019 | 0.334845 |
|  | \| sex*mean parental schooling*Tanner bre/gen \| \| --- \| | 0.0295 | 1 | 0.029516 | 0.011349 | 0.915371 |
|  | \| Error \| \| --- \| | 262.6729 | 101 | 2.600722 |  |  |

N.B. SS=sum of squares; DF=degrees of freedom; MS=mean sum of squares; F=F values; p=p values; SE=standard error of estimates (using sigma-restricted parameterization; effective hypothesis decomposition); PDS=Pubertal Development Scale scores (self-assessed); Tanner pubic and bre/gen=Tanner Pubic hair (both sexes) and breast (girls) and genital (boys) ratings by experienced clinicians, respectively; *=interactions.

Table 5S. Results of univariate general linear models regarding self-reported wake up times on weekends.

|  | Effects | \| SS \| \| --- \| | \| DF Freedom \| \| --- \| | \| MS \| \| --- \| | \| F \| \| --- \| | \| p \| \| --- \| |
| --- | --- | --- | --- | --- | --- | --- | --- | --- | --- | --- | --- |
| Age in in months (SE±1.900) | \| Intercept \| \| --- \| | 14.2383 | 1 | 14.23827 | 3.947872 | 0.049440 |
|  | \| sex \|  \| \| --- \| --- \| | 0.8286 | 1 | 0.82861 | 0.229751 | 0.632669 |
|  | \| age in months \| \| --- \| | 0.1291 | 1 | 0.12915 | 0.035809 | 0.850263 |
|  | \| mean parental schooling \| \| --- \| | 0.4284 | 1 | 0.42835 | 0.118771 | 0.731036 |
|  | \| sex*age in months \| \| --- \| | 0.1684 | 1 | 0.16843 | 0.046702 | 0.829309 |
|  | \| sex*mean parental schooling \| \| --- \| | 0.2911 | 1 | 0.29111 | 0.080717 | 0.776868 |
|  | \| age in months*mean parental schooling \| \| --- \| | 0.0500 | 1 | 0.05003 | 0.013873 | 0.906457 |
|  | \| sex*age in months*mean parental schooling \| \| --- \| | 0.0038 | 1 | 0.00383 | 0.001063 | 0.974047 |
|  | \| Error \| \| --- \| | 393.1160 | 109 | 3.60657 |  |  |
|  |  |  |  |  |  |  |
| PDS (SE±1.905) | \| Intercept \| \| --- \| | 61.0110 | 1 | 61.01097 | 16.80837 | 0.000080 |
|  | \| sex \| \| --- \| | 0.1739 | 1 | 0.17387 | 0.04790 | 0.827168 |
|  | \| mean parental schooling \| \| --- \| | 0.1541 | 1 | 0.15410 | 0.04245 | 0.837141 |
|  | \| PDS score \| \| --- \| | 0.3015 | 1 | 0.30146 | 0.08305 | 0.773751 |
|  | \| sex*mean parental schooling \| \| --- \| | 0.2585 | 1 | 0.25851 | 0.07122 | 0.790074 |
|  | \| sex*PDS score \| \| --- \| | 1.1365 | 1 | 1.13652 | 0.31311 | 0.576926 |
|  | \| mean parental schooling*PDS score \| \| --- \| | 0.4855 | 1 | 0.48554 | 0.13376 | 0.715270 |
|  | \| sex*mean parental schooling*PDS score \| \| --- \| | 1.2374 | 1 | 1.23740 | 0.34090 | 0.560517 |
|  | \| Error \| \| --- \| | 395.6478 | 109 | 3.62980 |  |  |
|  |  |  |  |  |  |  |
| Tanner pubic (SE±1.921) | \| Intercept \| \| --- \| | 94.6394 | 1 | 94.63943 | 25.62855 | 0.000002 |
|  | \| sex \| \| --- \| | 0.5879 | 1 | 0.58791 | 0.15921 | 0.690714 |
|  | \| mean parental schooling \| \| --- \| | 0.2372 | 1 | 0.23718 | 0.06423 | 0.800436 |
|  | \| Tanner pubic hair \| \| --- \| | 0.7581 | 1 | 0.75810 | 0.20530 | 0.651433 |
|  | \| sex*mean parental schooling \| \| --- \| | 0.1044 | 1 | 0.10443 | 0.02828 | 0.866779 |
|  | \| sex*Tanner pubic hair \| \| --- \| | 0.0055 | 1 | 0.00551 | 0.00149 | 0.969273 |
|  | \| mean parental schooling*Tanner pubic hair \| \| --- \| | 0.4736 | 1 | 0.47364 | 0.12826 | 0.720973 |
|  | \| sex*mean parental schooling*Tanner pubic hair \| \| --- \| | 0.1824 | 1 | 0.18236 | 0.04938 | 0.824581 |
|  | \| Error \| \| --- \| | 380.3516 | 103 | 3.69273 |  |  |
|  |  |  |  |  |  |  |
| Tanner bre/gen (SE± 1.945) | \| Intercept \| \| --- \| | 58.1875 | 1 | 58.18752 | 15.37641 | 0.000159 |
|  | \| sex \| \| --- \| | 0.1433 | 1 | 0.14327 | 0.03786 | 0.846107 |
|  | \| mean parental schooling \| \| --- \| | 3.0487 | 1 | 3.04873 | 0.80564 | 0.371504 |
|  | \| Tanner breast/genitalia \| \| --- \| | 2.2378 | 1 | 2.23782 | 0.59136 | 0.443655 |
|  | \| sex*mean parental schooling \| \| --- \| | 0.0001 | 1 | 0.00010 | 0.00003 | 0.995859 |
|  | \| sex*Tanner breast/genitalia \| \| --- \| | 0.0893 | 1 | 0.08931 | 0.02360 | 0.878205 |
|  | \| mean parental schooling*Tanner breast/genitalia \| \| --- \| | 2.0428 | 1 | 2.04283 | 0.53983 | 0.464172 |
|  | \| sex*mean parental schooling*Tanner bre/gen \| \| --- \| | 0.3618 | 1 | 0.36184 | 0.09562 | 0.757776 |
|  | \| Error \| \| --- \| | 389.7734 | 103 | 3.78421 |  |  |

N.B. SS=sum of squares; DF=degrees of freedom; MS=mean sum of squares; F=F values; p=p values; SE=standard error of estimates (using sigma-restricted parameterization; effective hypothesis decomposition); PDS=Pubertal Development Scale scores (self-assessed); Tanner pubic and bre/gen=Tanner Pubic hair (both sexes) and breast (girls) and genital (boys) ratings by experienced clinicians, respectively; *=interactions.

Table 6S. Results of univariate general linear models regarding estimated time in bed (TIB) on weekends.

|  | Effects | \| SS \| \| --- \| | \| DF Freedom \| \| --- \| | \| MS \| \| --- \| | \| F \| \| --- \| | \| p \| \| --- \| |
| --- | --- | --- | --- | --- | --- | --- | --- | --- | --- | --- | --- |
| Age in in months (SE±1.701) | \| Intercept \| \| --- \| | 26.5696 | 1 | 26.56961 | 9.118744 | 0.003152 |
|  | \| sex \|  \| \| --- \| --- \| | 0.6721 | 1 | 0.67213 | 0.230678 | 0.631984 |
|  | \| age in months \| \| --- \| | 0.0282 | 1 | 0.02816 | 0.009664 | 0.921869 |
|  | \| mean parental schooling \| \| --- \| | 0.5705 | 1 | 0.57053 | 0.195807 | 0.659003 |
|  | \| sex*age in months \| \| --- \| | 0.6729 | 1 | 0.67290 | 0.230942 | 0.631789 |
|  | \| sex*mean parental schooling \| \| --- \| | 1.2377 | 1 | 1.23774 | 0.424797 | 0.515925 |
|  | \| age in months*mean parental schooling \| \| --- \| | 0.0239 | 1 | 0.02390 | 0.008202 | 0.928002 |
|  | \| sex*age in months*mean parental schooling \| \| --- \| | 1.1083 | 1 | 1.10829 | 0.380368 | 0.538692 |
|  | \| Error \| \| --- \| | 317.5972 | 109 | 2.91374 |  |  |
|  |  |  |  |  |  |  |
| PDS (SE±1.687) | \| Intercept \| \| --- \| | 86.7476 | 1 | 86.74759 | 30.47137 | 0.000000 |
|  | \| sex \| \| --- \| | 3.6684 | 1 | 3.66840 | 1.28858 | 0.258800 |
|  | \| mean parental schooling \| \| --- \| | 1.2660 | 1 | 1.26604 | 0.44472 | 0.506264 |
|  | \| PDS score \| \| --- \| | 5.3013 | 1 | 5.30126 | 1.86215 | 0.175188 |
|  | \| sex*mean parental schooling \| \| --- \| | 2.7939 | 1 | 2.79385 | 0.98138 | 0.324052 |
|  | \| sex*PDS score \| \| --- \| | 6.5880 | 1 | 6.58797 | 2.31412 | 0.131099 |
|  | \| mean parental schooling*PDS score \| \| --- \| | 1.9561 | 1 | 1.95607 | 0.68710 | 0.408965 |
|  | \| sex*mean parental schooling*PDS score \| \| --- \| | 4.2058 | 1 | 4.20576 | 1.47734 | 0.226818 |
|  | \| Error \| \| --- \| | 310.3073 | 109 | 2.84686 |  |  |
|  |  |  |  |  |  |  |
| Tanner pubic (SE±1.637) | \| Intercept \| \| --- \| | 165.1094 | 1 | 165.1094 | 61.60728 | 0.000000 |
|  | \| sex \| \| --- \| | 1.5876 | 1 | 1.5876 | 0.59236 | 0.443268 |
|  | \| mean parental schooling \| \| --- \| | 10.4975 | 1 | 10.4975 | 3.91693 | 0.050471 |
|  | \| **Tanner pubic hair** \| \| --- \| | **18.2212** | **1** | **18.2212** | **6.79888** | **0.010476** |
|  | \| sex*mean parental schooling \| \| --- \| | 2.7250 | 1 | 2.7250 | 1.01679 | 0.315645 |
|  | \| sex*Tanner pubic hair \| \| --- \| | 4.3882 | 1 | 4.3882 | 1.63736 | 0.203563 |
|  | \| **mean parental schooling*Tanner pubic hair** \| \| --- \| | **12.0707** | **1** | **12.0707** | **4.50396** | **0.036214** |
|  | \| sex*mean parental schooling*Tanner pubic hair \| \| --- \| | 5.0903 | 1 | 5.0903 | 1.89933 | 0.171140 |
|  | \| Error \| \| --- \| | 276.0432 | 103 | 2.6800 |  |  |
|  |  |  |  |  |  |  |
| Tanner bre/gen (SE± 1.723) | \| Intercept \| \| --- \| | 96.3741 | 1 | 96.37414 | 32.45033 | 0.000000 |
|  | \| sex \| \| --- \| | 0.2526 | 1 | 0.25264 | 0.08507 | 0.771133 |
|  | \| mean parental schooling \| \| --- \| | 0.0026 | 1 | 0.00256 | 0.00086 | 0.976649 |
|  | \| Tanner breast/genitalia \| \| --- \| | 0.7769 | 1 | 0.77686 | 0.26158 | 0.610132 |
|  | \| sex*mean parental schooling \| \| --- \| | 0.2535 | 1 | 0.25348 | 0.08535 | 0.770761 |
|  | \| sex*Tanner breast/genitalia \| \| --- \| | 1.0341 | 1 | 1.03414 | 0.34821 | 0.556423 |
|  | \| mean parental schooling*Tanner breast/genitalia \| \| --- \| | 0.0170 | 1 | 0.01698 | 0.00572 | 0.939866 |
|  | \| sex*mean parental schooling*Tanner bre/gen \| \| --- \| | 0.6031 | 1 | 0.60307 | 0.20306 | 0.653208 |
|  | \| Error \| \| --- \| | 305.8994 | 103 | 2.96990 |  |  |

N.B. SS=sum of squares; DF=degrees of freedom; MS=mean sum of squares; F=F values; p=p values; SE=standard error of estimates (using sigma-restricted parameterization; effective hypothesis decomposition); PDS=Pubertal Development Scale scores (self-assessed); Tanner pubic and bre/gen=Tanner Pubic hair (both sexes) and breast (girls) and genital (boys) ratings by experienced clinicians, respectively; *=interactions; values in bold=p≤.05.

Table 7S. Results of univariate general linear models regarding self-reported Morningness-Eveningness Scale for Children (MESC) scores.

|  | Effects | \| SS \| \| --- \| | \| DF Freedom \| \| --- \| | \| MS \| \| --- \| | \| F \| \| --- \| | \| p \| \| --- \| |
| --- | --- | --- | --- | --- | --- | --- | --- | --- | --- | --- | --- |
| Age in in months (SE±5.318) | \| Intercept \| \| --- \| | 411.191 | 1 | 411.1915 | 14.54010 | 0.000226 |
|  | \| sex \| \| --- \| | 8.746 | 1 | 8.7456 | 0.30925 | 0.579259 |
|  | \| mean parental schooling \| \| --- \| | 45.645 | 1 | 45.6454 | 1.61406 | 0.206578 |
|  | \| age in months \| \| --- \| | 67.329 | 1 | 67.3292 | 2.38082 | 0.125679 |
|  | \| sex*mean parental schooling \| \| --- \| | 26.259 | 1 | 26.2595 | 0.92856 | 0.337332 |
|  | \| sex*age in months \| \| --- \| | 9.483 | 1 | 9.4826 | 0.33531 | 0.563720 |
|  | \| mean parental schooling*age in months \| \| --- \| | 49.380 | 1 | 49.3804 | 1.74614 | 0.189080 |
|  | \| sex*mean parental schooling*age in months \| \| --- \| | 30.922 | 1 | 30.9217 | 1.09342 | 0.297987 |
|  | \| Error \| \| --- \| | 3139.060 | 111 | 28.2798 |  |  |
|  |  |  |  |  |  |  |
| PDS (SE±5.331) | \| Intercept \| \| --- \| | 932.304 | 1 | 932.3043 | 32.80629 | 0.000000 |
|  | \| sex \| \| --- \| | 0.087 | 1 | 0.0867 | 0.00305 | 0.956042 |
|  | \| mean parental schooling \| \| --- \| | 55.401 | 1 | 55.4005 | 1.94946 | 0.165432 |
|  | \| PDS score \| \| --- \| | **109.427** | **1** | **109.4268** | **3.85055** | **0.052232** |
|  | \| sex*mean parental schooling \| \| --- \| | 4.099 | 1 | 4.0990 | 0.14424 | 0.704830 |
|  | \| sex*PDS score \| \| --- \| | 2.061 | 1 | 2.0614 | 0.07254 | 0.788177 |
|  | \| mean parental schooling*PDS score \| \| --- \| | 69.197 | 1 | 69.1966 | 2.43492 | 0.121507 |
|  | \| sex*mean parental schooling*PDS score \| \| --- \| | 11.163 | 1 | 11.1630 | 0.39281 | 0.532114 |
|  | \| Error \| \| --- \| | 3154.450 | 111 | 28.4185 |  |  |
|  |  |  |  |  |  |  |
| Tanner pubic (SE±5.457) | \| Intercept \| \| --- \| | 1041.991 | 1 | 1041.991 | 34.99604 | 0.000000 |
|  | \| sex \| \| --- \| | 14.896 | 1 | 14.896 | 0.50028 | 0.480942 |
|  | \| mean parental schooling \| \| --- \| | 35.951 | 1 | 35.951 | 1.20744 | 0.274353 |
|  | \| Tanner pubic hair \| \| --- \| | 68.955 | 1 | 68.955 | 2.31590 | 0.131063 |
|  | \| sex*mean parental schooling \| \| --- \| | 29.974 | 1 | 29.974 | 1.00668 | 0.318005 |
|  | \| sex*Tanner pubic hair \| \| --- \| | 23.187 | 1 | 23.187 | 0.77874 | 0.379543 |
|  | \| mean parental schooling*Tanner pubic hair \| \| --- \| | 46.839 | 1 | 46.839 | 1.57312 | 0.212539 |
|  | \| sex*mean parental schooling*Tanner pubic hair \| \| --- \| | 48.518 | 1 | 48.518 | 1.62952 | 0.204585 |
|  | \| Error \| \| --- \| | 3126.327 | 105 | 29.775 |  |  |
|  |  |  |  |  |  |  |
| Tanner bre/gen (SE± 5.219) | \| Intercept \| \| --- \| | 1287.847 | 1 | 1287.847 | 47.27775 | 0.000000 |
|  | \| sex \| \| --- \| | 2.857 | 1 | 2.857 | 0.10489 | 0.746678 |
|  | \| mean parental schooling \| \| --- \| | 68.677 | 1 | 68.677 | 2.52117 | 0.115334 |
|  | \| **Tanner breast/genitalia** \| \| --- \| | **120.538** | **1** | **120.538** | **4.42504** | **0.037804** |
|  | \| sex*mean parental schooling \| \| --- \| | 13.233 | 1 | 13.233 | 0.48580 | 0.487347 |
|  | \| sex*Tanner breast/genitalia \| \| --- \| | 7.972 | 1 | 7.972 | 0.29264 | 0.589678 |
|  | \| mean parental schooling*Tanner breast/genitalia \| \| --- \| | 82.664 | 1 | 82.664 | 3.03465 | 0.084432 |
|  | \| sex*mean parental schooling*Tanner bre/gen \| \| --- \| | 30.070 | 1 | 30.070 | 1.10388 | 0.295828 |
|  | \| Error \| \| --- \| | 2860.203 | 105 | 27.240 |  |  |

N.B. SS=sum of squares; DF=degrees of freedom; MS=mean sum of squares; F=F values; p=p values; SE=standard error of estimates (using sigma-restricted parameterization; effective hypothesis decomposition); PDS=Pubertal Development Scale scores (self-assessed); Tanner pubic and bre/gen=Tanner Pubic hair (both sexes) and breast (girls) and genital (boys) ratings by experienced clinicians, respectively; *=interactions; values in bold=p≤.05.

Table 8S. Results of univariate general linear models regarding estimated social jetlag.

|  | Effects | \| SS \| \| --- \| | \| DF Freedom \| \| --- \| | \| MS \| \| --- \| | \| F \| \| --- \| | \| p \| \| --- \| |
| --- | --- | --- | --- | --- | --- | --- | --- | --- | --- | --- | --- |
| Age in in months (SE±1.475) | \| Intercept \| \| --- \| | 1.3279 | 1 | 1.327907 | 0.610164 | 0.436374 |
|  | \| sex \| \| --- \| | 2.0873 | 1 | 2.087323 | 0.959110 | 0.329522 |
|  | \| mean parental schooling \| \| --- \| | 0.1581 | 1 | 0.158055 | 0.072625 | 0.788048 |
|  | \| age in months \| \| --- \| | 0.1225 | 1 | 0.122479 | 0.056278 | 0.812912 |
|  | \| sex*mean parental schooling \| \| --- \| | 0.5874 | 1 | 0.587402 | 0.269907 | 0.604419 |
|  | \| sex*age in months \| \| --- \| | 1.2308 | 1 | 1.230799 | 0.565543 | 0.453613 |
|  | \| mean parental schooling*age in months \| \| --- \| | 0.3153 | 1 | 0.315299 | 0.144878 | 0.704200 |
|  | \| sex*mean parental schooling*age in months \| \| --- \| | 0.1810 | 1 | 0.181043 | 0.083188 | 0.773556 |
|  | \| Error \| \| --- \| | 243.7471 | 112 | 2.176313 |  |  |
|  |  |  |  |  |  |  |
| PDS (SE±1.473) | \| Intercept \| \| --- \| | 3.9097 | 1 | 3.909714 | 1.802750 | 0.182095 |
|  | \| sex \| \| --- \| | 1.6518 | 1 | 1.651770 | 0.761623 | 0.384688 |
|  | \| mean parental schooling \| \| --- \| | 0.0004 | 1 | 0.000432 | 0.000199 | 0.988768 |
|  | \| PDS score \| \| --- \| | 0.2770 | 1 | 0.277029 | 0.127737 | 0.721463 |
|  | \| sex*mean parental schooling \| \| --- \| | 0.1428 | 1 | 0.142758 | 0.065825 | 0.797986 |
|  | \| sex*PDS score \| \| --- \| | 0.4152 | 1 | 0.415201 | 0.191447 | 0.662557 |
|  | \| mean parental schooling*PDS score \| \| --- \| | 0.0580 | 1 | 0.058013 | 0.026749 | 0.870378 |
|  | \| sex*mean parental schooling*PDS score \| \| --- \| | 0.0588 | 1 | 0.058753 | 0.027091 | 0.869561 |
|  | \| Error \| \| --- \| | 242.9000 | 112 | 2.168750 |  |  |
|  |  |  |  |  |  |  |
| Tanner pubic (SE±1.487) | \| Intercept \| \| --- \| | 1.3801 | 1 | 1.380105 | 0.623863 | 0.431379 |
|  | \| sex \| \| --- \| | 2.1610 | 1 | 2.161004 | 0.976860 | 0.325226 |
|  | \| mean parental schooling \| \| --- \| | 1.5014 | 1 | 1.501409 | 0.678697 | 0.411886 |
|  | \| Tanner pubic hair \| \| --- \| | 0.3823 | 1 | 0.382273 | 0.172803 | 0.678473 |
|  | \| sex*mean parental schooling \| \| --- \| | 0.5095 | 1 | 0.509491 | 0.230310 | 0.632283 |
|  | \| sex*Tanner pubic hair \| \| --- \| | 0.4976 | 1 | 0.497645 | 0.224955 | 0.636265 |
|  | \| mean parental schooling*Tanner pubic hair \| \| --- \| | 1.1388 | 1 | 1.138850 | 0.514806 | 0.474644 |
|  | \| sex*mean parental schooling*Tanner pubic hair \| \| --- \| | 0.0023 | 1 | 0.002335 | 0.001056 | 0.974141 |
|  | \| Error \| \| --- \| | 234.4926 | 106 | 2.212194 |  |  |
|  |  |  |  |  |  |  |
| Tanner bre/gen (SE± 1.464) | \| Intercept \| \| --- \| | 0.1786 | 1 | 0.178584 | 0.083283 | 0.773461 |
|  | \| sex \| \| --- \| | 3.0111 | 1 | 3.011080 | 1.404215 | 0.238669 |
|  | \| mean parental schooling \| \| --- \| | 4.9361 | 1 | 4.936092 | 2.301942 | 0.132189 |
|  | \| Tanner breast/genitalia \| \| --- \| | 2.1452 | 1 | 2.145198 | 1.000411 | 0.319489 |
|  | \| sex*mean parental schooling \| \| --- \| | 0.4909 | 1 | 0.490873 | 0.228918 | 0.633312 |
|  | \| sex*Tanner breast/genitalia \| \| --- \| | 0.9056 | 1 | 0.905636 | 0.422342 | 0.517176 |
|  | \| mean parental schooling*Tanner breast/genitalia \| \| --- \| | 4.2188 | 1 | 4.218813 | 1.967439 | 0.163642 |
|  | \| sex*mean parental schooling*Tanner bre/gen \| \| --- \| | 0.0071 | 1 | 0.007137 | 0.003329 | 0.954101 |
|  | \| Error \| \| --- \| | 227.2975 | 106 | 2.144316 |  |  |

N.B. SS=sum of squares; DF=degrees of freedom; MS=mean sum of squares; F=F values; p=p values; SE=standard error of estimates (using sigma-restricted parameterization; effective hypothesis decomposition); PDS=Pubertal Development Scale scores (self-assessed); Tanner pubic and bre/gen=Tanner Pubic hair (both sexes) and breast (girls) and genital (boys) ratings by experienced clinicians, respectively; *=interactions.
